# Bridging barriers, integrating insights: The Gotham approach to CTSA collaborative evaluation

**DOI:** 10.1017/cts.2025.10187

**Published:** 2025-11-03

**Authors:** Cathleen T. Kane, Elana E. Lipschitz, Zainab Abedin, Kawthar Muhammad, Brian J. Nickerson, Gina Rhim, Claudia Lechuga, Jonathan N. Tobin, Maija N. Neville-Williams, Chen Lyu, Alden Yuanhong Lai

**Affiliations:** 1 Clinical and Translational Science Institute, NYU Langone, New York, NY, USA; 2 Irving Institute for Clinical and Translational Research, Columbia University, New York, NY, USA; 3 ConduITS CTSA – Institutes for Translational Sciences, Icahn School of Medicine at Mount Sinai, New York, NY, USA; 4 Institute for Clinical and Translational Research, Einstein–Montefiore, Bronx, NY, USA; 5 Center for Clinical and Translational Science, The Rockefeller University, New York, NY, USA; 6 Clinical Directors Network (CDN), New York, NY, USA; 7 NYU School of Global Public Health, New York, NY, USA

**Keywords:** National Institutes of Health (NIH), multi-hub Clinical and Translational Science Awards (CTSA), translational science evaluation, evaluation study, stakeholder participation

## Abstract

Collaboration across the Clinical and Translational Science Award (CTSA) consortium is essential for advancing translational science, yet institutional silos often hinder data-sharing and benchmarking efforts. This study examines the viability of a voluntary, multi-hub analysis of the CTSA education common metric on trainee and scholar engagement across five New York City-based sites or “hubs.” Using a structured framework for collaboration and field-tested operational guidelines, a team of evaluators dubbed “The Gotham Group” pooled de-identified common education data to assess post-training research engagement and demographic representation. Their primary objective was to establish a sustainable model for independent data-sharing without national mandates or technical support. A secondary goal was to reassess the metric’s usefulness as an impact benchmark. Results showed that NYC education engagement percentages remained stable despite institutional differences, suggesting the metric’s viability for regional comparison. More importantly, the collaboration itself proved as valuable as its outcomes, fostering professional relationships, facilitating knowledge exchange, and strengthening evaluation capacity within and across the hubs. This study highlights the potential of voluntary data-sharing partnerships to overcome data silos and to create valuable networks driving continuous improvement in translational science.

## Introduction

Mutual collaboration is a cornerstone of the NIH-funded Clinical and Translational Science Award (CTSA) Program, a nationwide initiative to improve the efficiency and impact of clinical and translational research [1] administered by the National Center for Advancing Translational Sciences (NCATS). With over 60 CTSA hubs based in academic healthcare institutions, the program provides essential research services, training, and innovative approaches to accelerate the translation of scientific discoveries to improve human health. Given the complexity of this effort, rigorous and cooperative evaluation is essential [2]. This manuscript details the efforts of “The Gotham Group,” a voluntary partnership among a small group of evaluators from New York City-based CTSA sites or “hubs” testing the feasibility of independently pooling and analyzing regional education common metric data without national mandates or technical support. Beyond demonstrating a model for local data-sharing, the Gotham collaboration also strengthened evaluation capacity, fostered program innovation, and built a flexible yet durable professional network [3]. Its success lay not only in achieving its goals, but in showing that the collaborative process can be as valuable as the intended outcomes [4].

### CTSA evaluation

Since its inception in 2006, the CTSA Program has emphasized evaluation and tracking as central activities with an expanding mandate for continuous quality improvement and impact assessment. Every CTSA hub includes an evaluation team that collaborates closely with leadership and administration. In 2013, the Institute of Medicine (now the National Academy of Medicine) recommended the adoption of common metrics to evaluate and improve research activities across the consortium [5]. As a result, NCATS initiated the Common Metrics Initiative (CMI), with input from hub evaluators [6,7]. From 2015 to 2020, the initiative was managed centrally, with data collected from and reports delivered to all CTSA hubs annually. Although there were various concerns about metric design [8], ease of collection, and limited generalizability without supplemental data, the discontinuation of the CMI created a significant gap in cross-hub information. When the CMI ended, CTSA hub evaluators and leadership lost access to an essential set of consortium-specific benchmarks.

### The Gotham Group

In response to this development, a regional collective of evaluators came together from five New York City hubs (Columbia, Montefiore-Einstein, Mount Sinai, New York University, and Rockefeller) and dubbed themselves the “Gotham Group,” forming a self-directed community of practice aimed at regional evaluation collaboration [3,4]. The Gotham Group began planning their collaboration in 2019 soon after the first wave of CTSA Common Metrics were sunsetted. (CMI collection for two of the four CTSA Common Metrics ended in 2018; for the Education Metric, it ended in 2020.) The galvanizing questions at the heart of the Gotham Group project were simple: *Could this small group of hubs voluntarily replicate a previously nationally mandated and supported CMI evaluation activity at the regional level? And if yes, would the resulting benchmarks and the collaboration itself prove to be useful* [8]*?*

### Group purpose and project

The group decided to execute a small-scale evaluation project testing whether a regional hub could independently coordinate, collect, de-identify, and analyze data using the national CMI Operational Guidelines, without support from the NCATS Common Metrics Implementation Collaborative (CLIC). The CMI education metric (the percentage of CTSA trainees and scholars who remain engaged in research careers) was chosen as the initial proof-of-concept for its feasibility and relevance. The project also explored the benefits and challenges of collaboration among the participating New York City-based hubs, the feasibility of ongoing data collection, and the education common metric’s utility for local reporting and program improvement.

## Materials and methods

### Regional evaluation: How the Gotham Group was formed

The success of the Gotham Group’s evaluation depended entirely on voluntary collaboration. Members across a small set of local institutions (*N* = 5) established informal data-sharing agreements in a detailed group project plan created and continuously reviewed over the course of the process. To ensure rigor and relevance, the project was structured as a targeted evaluation and implementation science project to inform the broader CTSA consortium. The group convened regularly – initially in person and then virtually – to iteratively revisit the plan and monitor progress, securing approval from hub leadership at each participating institution. Of the six NYC hubs invited, only one declined to participate. After careful deliberation, the group chose to implement the previously mandated CMI Operational Guidelines for the education metric including key parameters and definitions such as:

- **Technical Description** (Including inclusion/exclusion criteria by role, specific parameters for “engaged in research,” as well as metric data timeframe and scope)
- **Metric Type** (Definition of the metric numerator and denominator, a continuous variable statement as well as inclusion/exclusion criteria for data based on: date ranges, training status, and/or trainees or scholars who have left the program or are currently in training.)
- **Data Sources and Methods of Collection** (Use of a single data collection template, e.g., rows and columns for common tracking sheets, the treatment of “lost to follow-up” data, the definition of a “program graduate,” and the range of acceptable data sources and systems – both manual and automated.)
- **Frequency of Data Collection and Reporting** (Data collection pegged to the calendar year)
- **Unit of Analysis** (KL2 and TL1scholars and trainees)

(For detailed description of the Education CMI Operational Guidelines, see Supplementary Material: SupplementaryMaterial_CCTR_Guideline.pdf).

These standardized preexisting guidelines kept the burden for participation manageable, ensured alignment with prior reports from NCATS/CLIC and promoted consistency in data collection across hubs, enhancing comparability and reliability [2]. In addition, utilizing a well-established and field-tested measure, rather than a novel one, helped ensure focus remained on the feasibility of collaboration rather than metric development. To safeguard confidentiality, an early consensus was reached that the hub-specific data collected for the project would not be shared across hubs within the group or any hubs external to the group. Instead, following the NCATS/CLIC reporting model, each hub would receive an individualized report summarizing its own data, presented in comparison to the new Gotham Group education engagement median percentage calculated over the study period. Recognizing the small sample size, the group chose not to subdivide the data by demographic variables such as gender, underrepresented groups in research, or hub size. These variations, however, were discussed collectively where they could provide deeper context to the evaluation findings at each hub.

### National common metric: % engagement of CTSA trainees and scholars over time

Following the specifications in the CMI Operational Guidelines to assess engagement in clinical and translational research among program graduates, the group first computed the percentage of engaged graduates within each participating hub. Engagement was defined as the proportion of program alumni who met the inclusion criteria and remained active in clinical and translational research. For each hub in the Gotham Group, the percentage (%) of graduates currently engaged in clinical and translational research was calculated as follows:

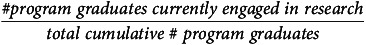

where the number of engaged graduates was divided by the total cumulative number of program graduates. The numerator included trainees (TL1) or scholars (KL2) who completed their program requirements in the time period specified in the CMI Operational Guidelines. The denominator included the cumulative total of TL1 or KL2 program graduates who met the inclusion criteria as stated in the CMI Operational Guidelines. This calculation and the inclusion/exclusion criteria were both structured in the same manner as the previously pooled consortium data: scholars and trainees who were still in training, left the program before completing requirements, or remained in residency or other degree-seeking programs were excluded from both the numerator and denominator.

### Gotham Group data collection and analysis

The CMI Operational Guidelines explicitly allowed flexibility in data collection methods, referencing acceptable techniques ranging from manual CV review to automated systems. As the Guidelines did not mandate a single data collection technique or platform across the more than 50 participating hubs, the Gotham Group (*N* = 5) maintained similar flexibility. In keeping with this approach, the project remained intentionally agnostic about how pooled data were collected. Following the established CMI Operational Guidelines, each participating hub de-identified scholar and trainee names, along with unique identifiers, before pooling their education common metric data. The NYU evaluation team consolidated these data into a master dataset, further anonymizing sub-level data by assigning each hub a non-identifying unique identifier. Once merged, the NYU CTSA biostatistician analyzed the dataset using standardized tables and visualizations from previous NCATS and CLIC reports. Engagement percentages were calculated for each of the five hubs, with current appointees and those lost to follow-up considered missing. Overall central tendency was estimated using the median to provide a robust measure of typical engagement while minimizing the influence of outliers. To ensure consistency and reproducibility, all calculations were conducted using R version 4.3.1. following standardized data processing protocols. Anticipating the potential need for future scalability, the analyst also developed a script for streamlined analyses in subsequent phases.

### Post-implementation facilitated discussions

As a final step in the process, the Gotham Group conducted facilitated discussions to critically assess the collaborative effort and its implications. These discussions aimed to revisit the objectives underlying the group’s formation and to document the hubs’ perspectives on the feasibility, utility, and challenges of an independent regional evaluation. Evaluators were asked to reflect on their experiences generating and using the metric as a group, particularly after NCATS/CLIC discontinued support. Specifically, these discussions and analysis examined: (1) the ongoing utility of the education common metric for local reporting and program improvement, as well as the perceived value of the pooled data; (2) the strengths and weaknesses of the metric itself in this context; (3) whether the collaboration was useful to participating hubs; (4) the barriers or limitations to collaboration. Discussions were held with each hub individually, as a group (i.e., each hub met virtually as a team and included all participating hub evaluators). An experienced qualitative researcher who was not involved in the hubs’ evaluation activities conducted the discussions with each hub using a semi-structured interview guide that was shared with participants in advance. The sessions were audio-recorded and transcribed. Qualitative coding focused on synthesizing perceived challenges, benefits, and areas for potential refinement. All evaluators from the Gotham Group reviewed the findings, which also provided context for interpreting the pooled data analysis, feasibility, and sustainability of the regional evaluation model.

## Results

### Evidence of engagement

The central indicator of the Gotham Group’s successful collaboration was the voluntary pooling of hub-level education data, as outlined in the CMI Education Operational Guidelines. Table 1 lists all participating NYC “Gotham” hubs along with the number of TL1 Trainees and KL2 Scholars reported by each for the time period outlined in the project plan. Variations in data volume across hubs reflect differences in award size (total funding) and program age (cumulative years since funding). Differences also exist within individual hubs. For example, Columbia and NYU reported nearly twice as many TL1 awards as KL2 awards, whereas Mount Sinai and Einstein had roughly equal numbers of each. Rockefeller, a smaller hub with a more focused scope, did not issue TL1 awards, but contributed KL2 data. Despite these variations, Table 1 confirms that all participating hubs aggregated data, with a total of 164 TL1 records and 133 KL2 records pooled. These data were also sufficient to calculate a regional median engagement rate with reasonable confidence levels.

**Table 1.** Total TL1 and KL2 graduates in pooled data set by Gotham Hub (2019–2021)

Total TL1 and KL2 graduates by Gotham Hub (2019–2021)		
**Gotham Hubs**	**Total TL1**	**Total KL2**
Columbia	60	34
Einstein	16	14
Mt. Sinai	29	33
NYU	59	26
Rockefeller	0	26
**Totals**	**164**	**133**

This table presents cumulative data from participating CTSA hubs on TL1 and KL2 graduates, including counts by year and program type. Data reflects aggregated reporting across hubs that participated in the Gotham Group initiative.

### Results of the pooled data

As a point of initial reference, Table 2 shows the pooled Education Common Metric data at the consortium level listed by year, median %, minimum and maximum %, and overall hub count for both the TL1 and KL2 data sets as it was conveyed to all CTSA hubs in November 2021 in the final CLIC Common Metrics report. As stated in the CMI Operational Guidelines, initial data collected in 2015 could reach back to trainees and scholars who received support as early as 2012, and the final data collected was pegged to the 2020 calendar year. Across the reporting years of 2015–2020, the consortium median % engaged for TL1s ranged from 85%–91% and the consortium median % engaged for the KL2s remained stable at 100% across all participating hubs.

**Table 2.** Consortium data 2015–2020: TL1 and KL2 graduates % engaged (median)

% TL1 graduates engaged (2015–2020) consortium medians						
**Year**	**2015**	**2016**	**2017**	**2018**	**2019**	**2020**
Data	**Consortium**					
Min	0%	0%	0%	0%	0%	0%
**Median**	**85%**	**87%**	**89%**	**88%**	**89%**	**91%**
Max	100%	100%	100%	100%	100%	100%
Hub count	46	50	50	50	49	48
**% KL2 graduates engaged (2015–2020) consortium medians**						
**Year**	**2015**	**2016**	**2017**	**2018**	**2019**	**2020**
Data	**Consortium**					
Min	0%	4%	0%	7%	78%	11%
**Median**	**100%**	**100%**	**100%**	**100%**	**100%**	**100%**
Max	100%	100%	100%	100%	100%	100%
Hub count	58	60	62	63	59	61

This table shows the percent of TL1 and KL2 graduates engaged in clinical and translational research activities across all CTSA Consortium hubs for all years that data was collected and reported on at the national level.

Table 3 below compares four years of Gotham regional data to the previous four years of consortium-wide data. Since the Gotham Group began in 2019 and completed their final data pull in 2023, the Gotham analysis covers the years 2019–2022. The group opted to use four years of data to stabilize the calculation of the median and to begin identifying trends. This also allowed them to incorporate previously submitted consortium data from 2019 and 2020, requiring only updated Gotham submissions for 2021 and 2022. Reading from left to right, Table 3 first presents consortium-level Education Common Metric data for 2015–2018, showing the same median %, minimum and maximum %, and total hub count for both TL1 and KL2 programs as listed in Table 2. The Gotham data appears in the second half of the table, showing the same summary metrics over time.

**Table 3.** Consortium vs. Gotham data 2015–2022: TL1 and KL2 graduates % engaged (median)

% TL1 Graduates enagaged consortium vs. Gotham								
**Year**	**2015**	**2016**	**2017**	**2018**	**2019**	**2020**	**2021**	**2022**
Data	**Consortium**				**Gotham Group**			
Min	0	0	0	0	88%	80%	75%	79%
**Median**	**85%**	**87%**	**89%**	**88%**	**94%**	**89%**	**83%**	**86%**
Max	100%	100%	100%	100%	100%	100%	93%	93%
Hub Count	46	50	50	50	4	4	4	4
**% KL2 graduates enagaged consortium vs. Gotham**								
**Year**	**2015**	**2016**	**2017**	**2018**	**2019**	**2020**	**2021**	**2022**
Data	**Consortium**				**Gotham Group**			
Min	0	4	0	7	81%	84%	88%	92%
**Median**	**100%**	**100%**	**100%**	**100%**	**100%**	**100%**	**100%**	**100%**
Max	100%	100%	100%	100%	100%	100%	100%	100%
Hub count	58	60	62	63	5	5	5	5

This table compares the percent of TL1 and KL2 graduates engaged in clinical and translational research activities across all CTSA Consortium hubs versus the median values for the Gotham Group subset.

For TL1 trainees, national consortium medians for engagement ranged from 85% to 89% between 2015 and 2018, based on data from approximately ∼50 hubs. In contrast, regional Gotham Group medians ranged from 83% to 94% between 2019 and 2022 with only four hubs (No Rockefeller hub data for TL1s). Although the Gotham TL1 medians were slightly more variable, they remained broadly consistent with national trends, especially considering sample size and disruptions like the COVID-19 pandemic. This suggests that, even with fewer contributing hubs, the regional median retained meaningful benchmarking potential. For KL2 scholars, the pattern was more pronounced. Nationally, the median engagement rate remained consistently at 100% from 2015 to 2020, with a hub count approximately ∼60 hubs. Similarly, in the Gotham dataset, the KL2 engagement median also held steady at 100% across the remaining years. The aggregated Gotham Group data offers key insights into the stability and limitations of utilizing the education metric at a regional scale. With only five hubs contributing data rather than the 50–60 hubs at the national level, the team expected significant variability and questioned whether the regional medians could serve as a reliable benchmark. However, Table 3 illustrates that despite the smaller sample size, the regional engagement medians remained relatively stable and, in some cases, very closely aligned with national trends.

### Themes of project post-implementation discussions

Integrating the qualitative perspectives of the Gotham Group members with the pooled common metric data provides a fuller picture of the feasibility, value, and limitations of regional evaluation efforts. Over the course of their discussions at the close of the project, a consensus emerged that the education common metric remained a useful, overarching indicator of training program impact. Additionally, all hubs reported that it served as a tool for continuous quality improvement enabling them to:

- Identify areas for expanded programmatic reach (e.g., specific research divisions, women scientists)
- Guide allocation of internal funding (e.g., seed grants)
- Support discussions with leadership on recruitment and mentorship
- Track longitudinal career progression
- Compare outcomes with peer hubs

Hub evaluators also frequently expressed the education common metric’s inability to capture the more nuanced and personalized nature of research careers both for trainees and scholars who were no longer engaged, as well as the individuals who remained engaged in clinical translational research. In spite of this tendency, the education common metric was also described as being useful operationally and strategically, since hubs were motivated to generate complementary data collection tools and/or potential modifications to inform and sustain its relevance.

Participating evaluators also uniformly reported that the Gotham Group collaboration had fostered a stronger professional network and their overall evaluation capacity including:

- A means of detecting and reviewing trends relevant to the region in a similar urban setting
- An enhanced ability to problem-solve and discuss common evaluation and program challenges
- A collective voice in data-driven conversations with institutional leadership with the advantage of information derived from their closest peers
- Leveraging the multi-hub collaboration in grant applications
- Receiving support during times of crisis (e.g., work changes due to the COVID-19 pandemic)
- The increased likelihood of future research collaborations

All Gotham Group members also acknowledged the time and effort required to sustain a voluntary regional community of practice as a major barrier – particularly for the organizer, who must coordinate and maintain ongoing engagement across hubs.

## Discussion

### Collaboration and common metrics: Both a means and an end

The Gotham Group and the education common metric both proved equally valuable as a means and an end. In terms of collaboration, the group successfully met their primary objective by independently pooling regional data without national mandates or external support. But in the process, a noteworthy additional benefit emerged: members began providing ongoing support beyond the project’s scope, sharing expertise in evaluation planning and grantsmanship while also serving as a professional network during challenges such as the pandemic, grant reapplications, and uncertainty surrounding National Institutes of Health (NIH) funding. Although the formal end goal was data aggregation and assessment, the greater achievement was breaking down institutional and professional silos among individuals who had been working in relative isolation, despite the geographical proximity. In terms of the education common metric, all participants (100%) reported continued usage for both internal and/or external reporting at their respective hubs. The project also helped overcome data silos, albeit regionally, that had reemerged after national reporting was no longer mandatory. Beyond its original purpose as a baseline measure, this metric also proved to be a means of driving internal program planning and continuous process improvement. In cases where the engagement metric was less than 100%, hub evaluators sought qualitative data to contextualize the findings. In some instances, data-driven decision-making led to new initiatives aimed at strengthening career engagement and support for trainees and scholars.

### Reflections on lessons learned

#### A valuable and lasting collaboration in a time of increasing uncertainty

In this multi-hub study, the collaboration among five NYC-based CTSA “Gotham” hubs was a defining feature and key strength. The findings highlight that replication of benchmarks is possible, and that the true value of multi-hub collaborations appears to lie not in replication per se, but in leveraging these kinds of collaborations to provide context, collegial feedback, insight, and action for local and national challenges. This function is more important than ever in a time of transition for the CTSA consortium.

#### Lessons regarding the education common metrics

Two key insights emerged from the evaluation of these metrics. First, despite the small number of contributing hubs, the engagement medians remained relatively stable, suggesting that regional evaluations can yield meaningful and potentially generalizable insights, albeit with the caution that the small sample size may limit the precision of these findings [8]. Second, there are limitations inherent in the design of the education common metric. (See Limitations below) This highlights the need for refinement to ensure the metric’s utility as a long-term benchmark [9,10]. Several revisions to the education common metric are proposed. These include but are not limited to: (1) Revising the cumulative denominator; (2) Refining the operational definition of “engaged” to better capture the diverse ways in which trainees and scholars contribute to clinical and translational research; and (3) Integrating qualitative data to provide a richer, more contextually informed evaluation [11].

#### Lessons on regional collaboration

The original goal of this study was to assess whether CTSA evaluators could independently organize a collaborative evaluation effort without the external facilitation and support previously provided by NCATS/CLIC. The Gotham Group proof-of-concept also demonstrated how regional collaboration can foster knowledge exchange and generate organizational benefits, including improved problem-solving and a collective voice in institutional dialog. The innovative aspect of this effort ultimately lay in the collaborative model itself, marking it as a noteworthy exemplar of a decentralized, self-directed, and regional multi-hub evaluation effort in the absence of centralized oversight. However, while the project demonstrated feasibility and benefits, self-directed collaborations require participating hubs to allocate time and resources without external incentive structures. Ensuring sustainability and scalability depends on identifying shared incentives and expanding participation – challenges that warrant further exploration.

### Implications for future collaborations and regional evaluation

#### An ongoing need for benchmarks

The initial goal of replicating an established benchmark was successfully achieved by the Gotham Group, but the process of generating this data illuminated several challenges related to the effective use of such benchmarks in settings with limited participation (*N* = 5). As newer data-sharing platforms such as *Flight Tracker* (a REDCap tool to streamline career development grant preparation and reporting) [12] and evaluation frameworks like the *Translational Science Benefits Model* [13] gain traction within the CTSA network, the role of communities of practice such as the Gotham Group, may become increasingly critical. These communities can provide the qualitative context and facilitate rapid, expert feedback necessary to enhance ongoing evaluations and decision-making. Furthermore, the national CTSA evaluation community continues to express an appetite for usable national benchmarks. Regular internal surveys [14] of national evaluators with >90% response rates report that 42% of current hubs continue using the common metrics in some form. In 2025 [15], a national evaluation study summarized a comprehensive set of measures for evaluating the central goals of the CTSA Program based on input from over 40 hubs and more than 100 key stakeholders including CTSA Administrators, CTSA Evaluators, and NCATS staff. The education common metric (and other CMI benchmarks) remained in this data set of >80 suggested measures.

#### Evaluation communities of practice

As hubs continue to utilize the common metrics independently, broader participation and data benchmarks across the consortium will be essential to establishing reliable common metrics going forward. Moreover, these findings suggest that alternative benchmarking tools may also emerge as more viable options for future evaluation efforts, especially in the absence of centralized infrastructure. The true value of communities of practice such as the Gotham Group [16–18], ultimately lies in their ability to provide timely, expert feedback that can inform local decision-making [19] and the broader advancement of translational science [20].

#### Limitations of the methods and the context for collaboration

This collaboration and analysis, while providing valuable insights, is subject to several limitations that must be considered. The relatively small number of participating hubs (five in the regional group compared to the greater than 60 in the national consortium) raises questions about potential inconsistencies in reporting and/or biases in data interpretation, as well as the generalizability of the findings and the precision of the estimates. Since all Gotham scholars and trainees were based at urban hubs in New York City, their percent engagement findings may not generalize to more rural or other varied contexts. Also, the stability of the median values may obscure underlying variability across individual NYC hubs whose institutional resources, variation in evaluator roles and hub capacity differ significantly despite the geographic proximity. Additional limitations stem from the structure of the Education CMI Operational Guidelines, and while these remain noteworthy, they are not unique to the Gotham project. The Guidelines define inclusion and exclusion criteria and provide a standardized reporting template but offer limited guidance for real-world gaps – such as years without graduates or ambiguous engagement status. They also allow flexibility in local data collection techniques, interpretation and data systems which may lead to variation in data quality. The metric also compares a point-in-time numerator (“currently engaged”) to a cumulative denominator (“cumulative number of graduates”) that grows indefinitely. As earlier cohorts retire, the engagement rate may decline even if recent alumni remain active. Over time, the structure of this metric risks reflecting cohort aging and mathematical trends more than true program impact. For instance, the KL2 engagement percentage will not indefinitely remain at 100%, and this decline could reflect natural factors outside of the guideline definition for engagement. Changes to the Education Operational Guidelines such as annualizing the denominator in lieu of the current culminative denominator could ameliorate these methodological limitations and improve the utility of this metric.

In terms of the collaboration itself, the scalability of this initiative to include a broader sample of hubs would introduce significant logistical and resource-related challenges, making it difficult to expand this model without additional support and coordination at the national level. This particular collaboration was also impacted by several acute external factors. The group was initially launched in early 2019. Shortly thereafter, the COVID-19 pandemic hit, presenting well-documented challenges [21] to project continuity and the nature of group collaboration, not to mention the research, education and training programs taking place at that time. At different points throughout the course of the project, every participating hub also underwent the process of reapplying for CTSA funds, a process that takes well over a year and draws heavily on evaluation staff and resources. Finally, at the time of this writing the CTSA is experiencing an unprecedented moment of uncertainty regarding the future of the NIH [22], NCATS as an agency and the very existence of the CTSA grant. These external limitations are not immaterial and constitute serious limitations in the context of this collaborative evaluation effort.

## Conclusion

What makes this collaboration remarkable is that it persisted, and may have even been strengthened, precisely because of the significant external limitations it faced. The disruptions caused by the COVID-19 pandemic, the resource-draining process of CTSA renewal, and the broader uncertainty surrounding the future of NIH and CTSA funding were not minor setbacks; they were fundamental challenges that could have easily derailed the group’s efforts. These obstacles were not merely logistical hurdles but existential threats to the continuity of the project, affecting institutional priorities, funding structures, and the capacity of individual hubs to engage fully. Instead, these constraints became a proving ground for the group’s resilience. Rather than stalling, the collaboration evolved, sustained momentum, and arguably gained new relevance and urgency including the ability to continue to produce meaningful work. The Gotham Group was not only a success because of what it did: a useful proof-of-concept on the feasibility and replicability of a pooled dataset of common metrics; but also, because of what it did not do: dissolve in the face of profound uncertainty. To view this collaboration solely through the lens of its output would be to overlook a critical part of its story. The true measure of its success lies not only in what it accomplished but also in the limitations it overcame. This group did not merely persist, it demonstrated, in real-time, the essential value of its work by proving that collaboration, even under extreme constraints, is not just possible but necessary.

## Supporting information

10.1017/cts.2025.10187.sm001 Kane et al. supplementary material Kane et al. supplementary material

## Supplementary material

The supplementary material for this article can be found at https://doi.org/10.1017/cts.2025.10187.
