# Supplementary material for: Bridging barriers, integrating insights: The Gotham approach to CTSA collaborative evaluation
Source: J Clin Transl Sci. 2025 Nov 3;9(1):e262. doi: 10.1017/cts.2025.10187 (PMC12766516; doi:10.1017/cts.2025.10187)
Supplement: Kane et al. supplementary material [file S2059866125101878sup001.pdf]

CTSA Common Metric (CM) Operational Guideline: *Careers in Clinical and Translational Research*

**Template Element**	**Description**
1. Operationalized Metric Title	Clinical and Translational Research Career Outcomes
2. Rationale	The purpose of this metric is to measure the outcomes of TL1 Trainees and KL2 Scholars and to use these data to develop strategic management plans that enhance the effectiveness of CTSA career development programs
3. Operational Specification	The measurement is the number and percent of a hub's Scholars and Trainees who completed the KL2 and TL1 (pre- and/or post-doc) program requirements, and who are currently engaged in clinical and translational research. Of those who are currently engaged in clinical and translational research, the specific assessment is the number and percent who are:<br>• Females<br>• Males<br>• Other (transgender, gender non-binary, intersex, etc.)<br>• Underrepresented persons<br>• Underrepresented persons who identify as female<br>• Underrepresented persons who identify as male<br>• Underrepresented persons who identify as other (transgender, gender non-binary, intersex, etc.)
4. Technical Description	**Key Definitions:**<br>• The TL1 program is also known as the National Research Service Award (NRSA) Training Core.<br>• The KL2 program is also known as the Institutional Career Development Core.<br>• NIH definition for underrepresented persons includes individuals from underrepresented racial and ethnic groups, individuals with disabilities, individuals from disadvantaged backgrounds and females at senior faculty levels in biomedical-relevant disciplines.<br>○ https://grants.nih.gov/grants/guide/notice-files/NOT-OD-20-031.html (Release Date: November 22, 2019)<br>• **"Engaged in research"** includes any of the following activities:<br>○ If the primary role is as a **clinician**: some effort is spent engaged in research or as a site PI for industry-sponsored clinical trials or being an active member of a multidisciplinary research team<br>○ If the primary role is in **academia**: PI, Co-PI, or Co-I on a funded clinical and translational research project<br>○ If the primary role is in **academia** and is actively involved in a project writing/re-submitting/reviewing clinical and translational research<br>○ If the primary role is in **industry**: involved in directing clinical and translational research projects

	○ If the primary role is in **government**: involved in conducting or managing clinical and translational research<br>○ If the primary role is at a **foundation**: involved in funding, conducting, or managing clinical and translational research<br>○ If the primary role is in a **private organization**: involved in funding, conducting, or managing clinical and translational research<br>**Timeframe:**<br>This metric will be collected annually (calendar year) and is cumulative for all KL2 Scholars and TL1 Trainees who completed their program requirements since January 1, 2012 (even if appointed before this date). If the hub was funded later than this date, the beginning date for data collection and reporting is as of the initial funding date.<br>**Data Scope:**<br>All Scholars and Trainees who completed their TL1 or KL2 program requirements after January 1, 2012, respectively. TL1 data is reported separately from KL2. Program graduates for whom data cannot be obtained are not included in the numerator (# program graduates engaged in research) or in the denominator (total # program graduates).
5. Metric Type, Score(s), Numerator and Denominator Statements or Continuous Variable Statement, Inclusion/Exclusion Criteria	This metric results in the following scores (A-C) for each program (KL2, TL1-predoc, and TL1-postdoc, as applicable):<br>**A.** Number of prior (KL2 or TL1) appointees who are currently engaged in clinical and translational research.<br>**B.** Percent of prior (KL2 or TL1) appointees who are currently engaged in clinical and translational research.<br>**C.** Of the prior (KL2 or TL1) appointees who are currently engaged in clinical and translational research, the number and percent of underrepresented persons, stratified by:<br>○ Male<br>○ Female<br>○ Other (transgender, intersex, gender non-binary, etc.)<br>***A. Number of prior (KL2 or TL1) appointees who are currently engaged in clinical and translational research*.**<br>**Metric Type:** Count<br>• **Inclusion Criteria:** The total (cumulative) number of (KL2 or TL1) program graduates since January 1, 2012, who are currently engaged in clinical and translational research. This also includes Scholars and Trainees who receive a new career development award.<br>• **Exclusion Criteria:** Scholars and Trainees who are still in training or who have left the program without completing the full training program requirements. Program alumni who are still in residency or other degree-seeking programs are also excluded.

	B. Percent (%) of graduates who are currently engaged in clinical and translational research<br>**Metric Type:** Rate<br>*Denominator Statement: The total (cumulative) number of (KL2 or TL1) program graduates since January 1, 2012.*<br>• **Inclusion Criteria for Denominator:** The total (cumulative) number of (KL2 or TL1) program graduates since January 1, 2012, who are currently engaged in clinical and translational research. This also includes Scholars and Trainees who receive a new career development award.<br>• **Exclusion Criteria for Denominator:** Scholars and Trainees who are still in training or who have left the program without completing the full training program requirements. Program alumni who are still in residency or other degree-seeking programs are also excluded.<br>*Numerator Statement: The numerator for this score is the result for* ***score A (above)***<br><br>***C. The number of prior male, female, and other KL2 and/or TL1 appointees who are:***<br>• Underrepresented persons<br>• Non-underrepresented persons<br>• Underrepresented persons engaged in research<br>• Underrepresented persons not engaged in research<br>• Non-underrepresented persons engaged in research<br>• Non-underrepresented persons not engaged in research<br>**Metric Type:** Count<br>*Provide data for KL2, TL1 pre-docs, and TL1 post-docs separately.*
6. Data Sources & Methods of Data Collection	**Data Collection Template & Guidance**<br>Hubs should use the template provided below (also available on CLIC website) to collect the raw data needed for common metrics reporting and internal strategic planning. Collecting data in this format will allow hubs to track engagement in research over time by Scholar/Trainee.

~ Data Collection Template ~

Last Name	First Name	Program (1=KL2, 2=TL1-postdoc, 3=TL1-predoc)	Year Started	URP (1=yes, 0=no)	Gender: Male/Female/ Other (1=M, 2=F, 3=Other)	Engaged in Research Status by Year (0=non engaged, 1=engaged, 2=current appointee, 3=lost to follow up)			
						2019	2020	2021	2022

	**Definitions:**<br>• "Engaged in research" as defined in **Technical Description** section above<br>• "Lost to follow-up" designation is for any former Scholar/Trainee for whom their current research status cannot be determined. Hubs should continue to try to follow-up with this Scholar/Trainee for 5 years before dropping them from the dataset. Those who are lost to follow up after 5 years, are not included in the denominator, for common metrics reporting but hubs should continue to try to track them, as they may re-enter clinical and translational research.<br>• "Program graduate"<br>○ Trainees who have completed all of their program requirements<br>○ Trainees who leave the program after receiving an F/K award<br>○ Trainees who leave the program to accept a research position<br>**Potential Data Sources:**<br>• Current year CVs of Scholars/ Trainees (requested annually by program administrator). Manual effort reading CVs. May require back-up data collection via internet searches and/or contacting former Scholars/Trainees.<br>• Survey application (e.g. Graduate Tracking Survey System - GTSS - developed by Rockefeller University; WebCAMP PROMPTR- developed by Weill Cornell University; Flight Tracker for Scholars - Vanderbilt University) or a survey developed in-house at a CTSA Program hub.<br>**Note**: NIH Reporter only reports PIs and not Co-PIs, so it cannot be the sole source of information for NIH grants, particularly for team science, where involvement may be as a team member but not as PI.
7. Frequency of Data Collection and Reporting	Hubs will aggregate data annually (January 1 - December 31). Data is entered in the CM-PRISM software application on the last business day of August each year.<br>**One** Program Summary is required from each hub for the Careers metric. Hubs that are funded for both KL2 and TL1 grants can select which metric to use for the Program Summary.
8. Unit of Analysis	Scholar (KL2), Trainee (TL1)
